# Optical Studies of Thin Films of Cryocondensed Mixtures of Water and Admixture of Nitrogen and Argon

**DOI:** 10.3390/ma15217441

**Published:** 2022-10-24

**Authors:** Dmitriy Y. Sokolov, Darkhan Yerezhep, Olga Vorobyova, Miguel A. Ramos, Ainura Shinbayeva

**Affiliations:** 1 Department of Thermal Physics and Technical Physics, Al-Farabi Kazakh National University, Al-Farabi Av. 71, Almaty 050040, Kazakhstan; 2 Department of Machines and Devices of Manufacturing Processes, Almaty Technological University, Tole Bi Av. 100, Almaty 050012, Kazakhstan; 3 Departamento de Física de la Materia Condensada, Instituto “Nicolás Cabrera”, Universidad Autónoma de Madrid, E-28049 Madrid, Spain; 4 Centro de Micro-Análisis de Materiales (CMAM), Campus de Cantoblanco, Universidad Autónoma de Madrid, E-28049 Madrid, Spain

**Keywords:** cryocondensation, glass transition, IR spectrometry, PVD, argon, nitrogen, supercooled thin films

## Abstract

The interaction of host molecules with water molecules is of primary importance in astrophysical and atmospheric studies. Water-binding interactions continue to attract a broad interest in various fields, especially those related to the formation of assembly structures. Using the physical vapor deposition (PVD) method and a two-beam interferometer with a wavelength of 406 nm, the refractive indices of thin films of a water and nitrogen (argon) mixture were calculated in the range from 15 to 35 K. The results of temperature transformations of the obtained films from a two-beam interferometer, and thermal desorption characteristics from the temperature of condensation to the temperature of evaporation of water (15–180 K), are presented. The relationship between the signal of the interferometer, the refractive index, and the film thickness during glass transition is demonstrated.

## 1. Introduction

The increased interest in molecules that are cold enough to form ice has led to a wealth of useful information on various properties. The review of articles helps to understand the significance of the studied parameters (such as refractive index, density, and polarizability) of amorphous structures at low temperatures obtained in the experiment. At present, various methods have been described for studying the refractive index for a wide range of substances [1], at low temperatures, including for substances such as freon [2]. 

The gradual and reversible transition in amorphous structures (or the presence of amorphous structures inside semi-crystalline materials) from a solid and relatively brittle state to a viscous or elastic state with increasing temperature is called the glass transition process. In nature, clathrates and other amorphous structures such as glass are often present in sediments containing both natural gas and water. Clathrates are widely distributed in permafrost regions and in thermodynamically stable regions of the ocean, as well as in interstellar space, and therefore are important gas reserves. The appearance of clathrates in liquid inclusions was first described by Roedder in 1963 [3].

Unlike [4], we used FTIR to examine the behavior of each molecular vibration during glass transition. Changes in the absorption spectrum of FTIR hydrogen bond vibrations, including in mixtures, were previously studied by different authors [5,6,7]; in recent works [8] one can find the effect of the plasma frequency on the formation of clathrate compounds and changes in the FTIR-ATR spectrum. We wanted to focus on finding vibrations that reflect delocalization in a clathrate or any other structure capable of keeping a guest molecule halfway to a glass transition or crystallization of the host molecule.

At present, interest in the properties of mixtures at low temperatures with substances such as water [9,10,11,12] and alcohols [13,14,15,16] remains relevant. In addition to experimental works, a large number of works are devoted to calculations [16]. 

To study the optical properties of thin films of cryocondensed mixtures, the following problems were formulated:

Firstly, what structures are formed during the glass transition of mixtures of water and various gases (such as nitrogen, argon, methane, carbon dioxide, etc.).

Secondly, what types of hydrogen bond vibrations occur during the interaction of a guest molecule and a host molecule of an amorphous structure.

Thirdly, how does the concentration of water in a mixture of water and nitrogen/argon affect the optical properties of the IR spectrum.

The aim of this work is to study the effect of gaseous impurities on the dependence of the refractive indices and on structural-phase transitions inside the sample during heating. We carried out several cycles of experiments with mixtures of water, methanol, and ethanol with nitrogen and argon. In this work, we begin with a detailed description of the study of thin films of solid water with nitrogen and argon obtained in the temperature range from 15 to 35 K.

Simple molecules such as H_2_, N_2_, CO, Ar, and CH_4_ that have been adsorbed in amorphous or crystalline ice are often used as an IR-mark to study the properties of ice and glass. When water molecules dissolve in matrices of simple molecules at low temperatures, very clear discrete bands of monomeric, dimeric, and polymeric water appear. The infrared spectrum of water, in various solid matrices of simple molecules, has been the subject of several studies [17,18,19,20,21,22,23].

**Figure 1:**
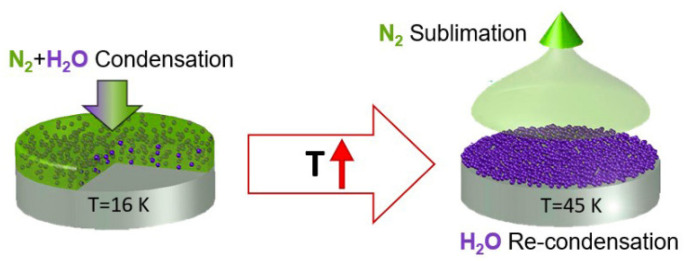
The scheme of the process of recondensation.

The components of ice, which are mixtures of different gases, have different sublimation temperatures, which, when the temperature rises, can lead to drastic changes in their structure. For example, for a two-component sample, when the temperature reaches above the sublimation temperature of one of the components of the mixture, it will evaporate, resulting in recondensation of the second component on the substrate. A schematic diagram of the recondensation process is shown in Figure 1.

Our studies have shown that the properties of recondensates, including optical ones, differ significantly from the properties of single-component thin films. Thus, the recondensation process will be radically different from classical physical vapor deposition (PVD), because in the recondensate, the structure will be affected not by individual gas molecules, but by its polyaggregates, which were previously formed in the film during the condensation of the initial two-component sample [24]. This is followed by the scientific significance of the experiments carried out reflected in this manuscript: the addition of information about the properties and behavior of recondensates of various gases obtained from various mixtures. This also complements the verification databases used to interpret astrophysical observations, determine the composition of space ice, and study greenhouse gases in ocean ice.

In this work, we study thin films of mixtures of water with argon and nitrogen and their properties during structural-phase transformations. What follows is a description of the experimental methodology and setup, followed by a description of the results that were obtained during the study.

## 2. Materials and Methods

### 2.1. Materials

Three-times-distilled water, argon (highest purity grade (99.993%) argon gas with maximum oxygen fraction not exceeding 0.0007%, water vapor not exceeding 0.0007%, and containing no more than 0.0005% of nitrogen (ISKHAN TEHNOGAS LLP, Almaty, Kazakhstan) GOST 10157-79), and nitrogen (first grade nitrogen (99.999% purity) with maximum oxygen fraction not exceeding 0.0005% and water vapor not exceeding 0.0007% (ISKHAN TEHNOGAS LLP, Almaty, Kazakhstan) GOST 9293-74) were used as the test substances. The purity of chemicals was evaluated by measuring their saturated vapor pressure at a temperature of 293.15 K and comparison with literature values. 

### 2.2. Experimental Methodology

**Figure 2:**
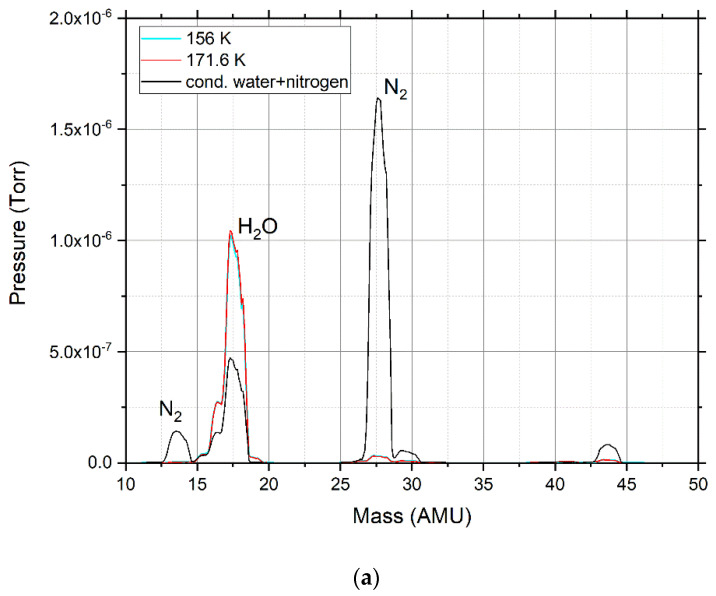

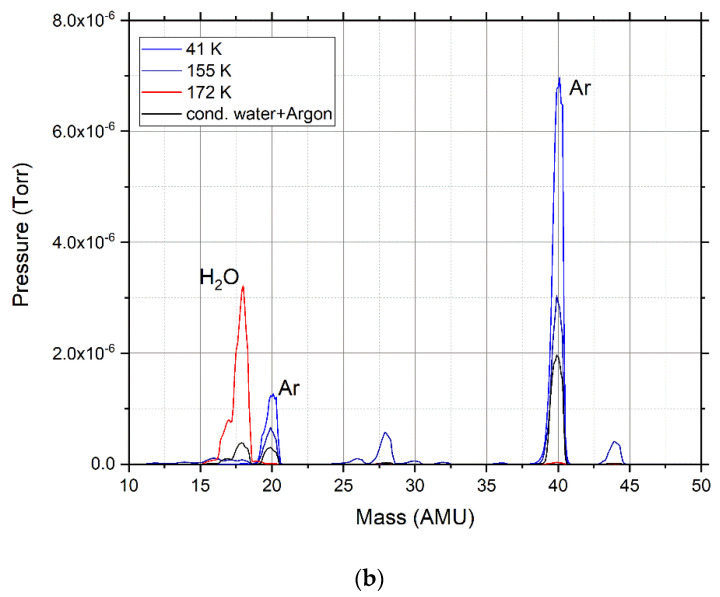
Mass spectra of condensation mixtures: (**a**) water and nitrogen; (**b**) water and argon.

The mixtures of the systems under study were freshly prepared by mixing carefully selected volumes of pure liquids and gases at a temperature of ~293 K in the inlet system volume. The mixtures were mixed uniformly. Extreme care was taken to minimize preferential evaporation during the filling process by creating the mixture at pressures below saturated vapor pressures. During gas puffing into the chamber and condensation, the mass spectrum of the mixture was measured using an Extorr XT Series RGA Model XT100 (Extorr Inc., New Kensington, PA, USA). Figure 2a shows the mass spectrum of the 25 percent concentration of water and nitrogen. Figure 2b shows the mass spectrum the 25 percent concentration of water and argon.

### 2.3. Experimental Setup

**Figure 3:**
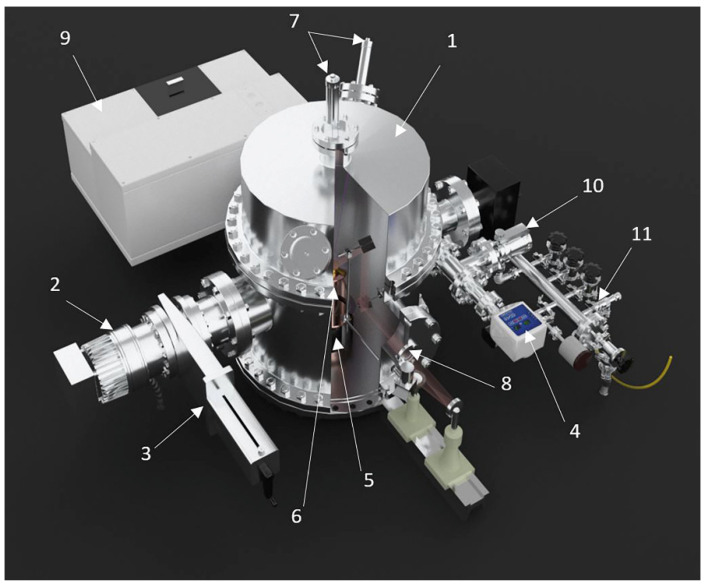
Experimental setup for cryovacuum condensation: 1: vacuum chamber, 2: vacuum pump Turbo-V-301, 3: vacuum gate valve CFF-100, 4: pressure detector FRG-700, 5: Gifford–McMahon refrigerator, 6: substrate, 7: photo multiplier, laser interferometer, 8: light source, optical channel, 9: IR-spectrometer, 10: high-precision gas supply leak into the chamber; 11: gas leak into the mixture production system.

The main unit of the experimental setup (see Figure 3) is a high vacuum chamber which generally works at 0.01–1 μTorr. The continuous pumping process is carried out by a Turbo-V- turbomolecular pump (Agilent, Santa Clara, CA, USA) in combination with an SH-110 dry-scroll vacuum pump (Agilent, Santa Clara, CA, USA). Pressure measurement is carried out by the FRG-700 converter (Agilent, Santa Clara, CA, USA) with an AGC-100 controller (Agilent, Santa Clara, CA, USA).

Inside the vacuum chamber, the substrate is in thermal contact with a closed-cycle helium Gifford–McMahon refrigerator, due to which the temperature can be varied in the range of 12–200 K. This copper substrate, 60 mm in diameter (6), is covered with a flat silver film. Temperature control is carried out by a silicon diode TS 670–1.4 connected to an M335/20 thermostat, which maintained the temperature constant with an accuracy of 0.5 K. A two-stage cooling system cooled the substrate to 12 K. A resistor heater was connected to the end of the second stage.

The Extorr XT100, mounted in a vacuum chamber, is a quadrupole residual gas analyzer that also includes an advanced Pirani probe as well as a hot cathode Bayard/Alpert (B/A) ion probe. The Pirani pressure gauge measures vacuum based on the thermal conductivity of the gaseous medium. The B/A ion sensor uses electron impact ionization of residual gases to measure pressure-related ion current. The quadrupole gas analyzer uses precision mechanics and electronics to measure the ion currents due to the partial pressures of the residual gases in the vacuum chamber. Pirani starts at atmospheric pressure, B/A ionometer at 10 mTorr nominal pressure, and quadrupole starts at 0.1 mTorr nominal pressure. The XT100 operates from 1 to 100 amu.

**Figure 4:**
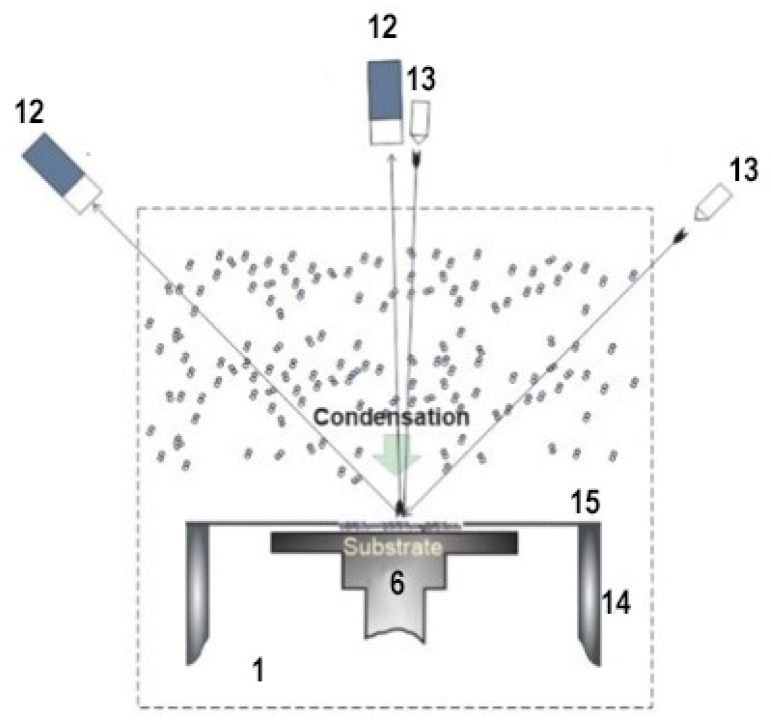
Experimental setup for laser-beam patterning during cryovacuum condensation: 6: substrate, 12: photoelectric multiplier, 13: laser, 1: vacuum chamber, 14: metal cylinder, 15: shield. Number labelling follows that of Figure 3. Reproduced with permission from A.U. Aldiyarov, Refractive Index at Low Temperature of 478 Tetrachloromethane and Tetrafluoroethane Cryovacuum Condensates; published by ACS Omega, 2020 [2].

A special protective screen (see Figure 3 and Figure 4) ensures the deposition of all the injected gas on the substrate when the pumping of the vacuum chamber is stopped. Using a metal cylinder (13) and a screen (14), it is possible to isolate the substrate (6) and all cold elements of the microcryogenic device from the main volume of the vacuum chamber.

This insulation is leaky, but at operating pressures of 1–10 µTorr it is quite effective because the mean free path of molecules at these pressures is several orders of magnitude greater than the distance between the protective screen and the substrate. Therefore, during our experiments, all the gas is deposited on the substrate when the pumping line is closed. The mixture of water and nitrogen (argon) is pre-uniformly mixed in the mixture production system (11). Then the homogeneous mixture is fed into the cryovacuum chamber for condensation.

Figure 4 shows an experimental setup for imaging a laser beam when a mixture sample is condensed onto a camera substrate. Two laser beams pass through the optical drives (7), and both beams converge at the center of the substrate. After reflection, the signal from the laser (13) arrives at two P25a-SS-0-100 photomultipliers (12).

**Figure 5:**
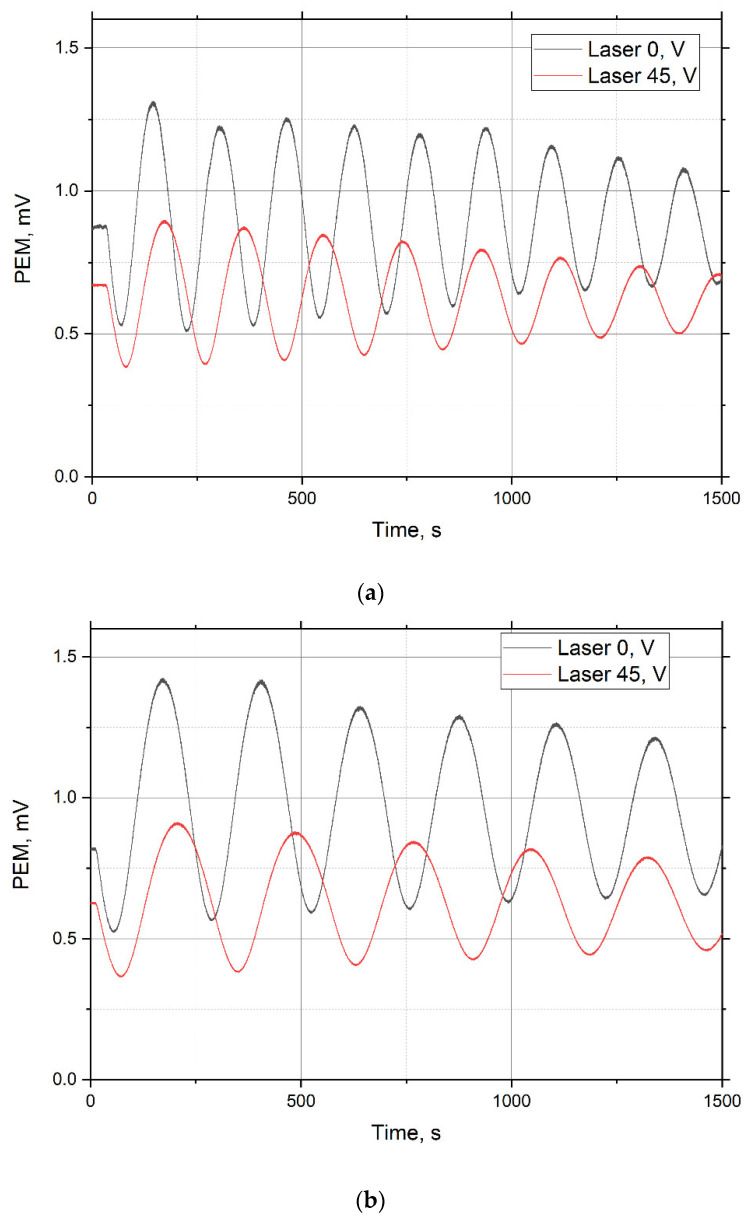
Interference patterns during the deposition of mixture of water and nitrogen (**a**) and mixture of water and argon (**b**). α_1_ = 1°, α_2_ = 45°.

In Figure 5, emitted photoelectron interference patterns (PEM) are generated by a diode laser and two photomultipliers. The measurements were carried out at a frequency of 100 Hz, which makes it possible to determine the oscillation period with an accuracy of ±0.05 s. Then the thin cryofilm thickness d and its refractive index n were measured.

Condensation of the water-nitrogen and water-argon mixtures took place at a temperature of 16 K, and the thickness of the cryofilms was the same and amounted to 3.9 μm.

The refractive index is calculated by the following Equation (1):

(1) $$n=\sqrt{\frac{t_{2}^{2}\sin^{2}\alpha_{2}-t_{1}^{2}\sin^{2}\alpha_{1}}{t_{2}^{2}-t_{1}^{2}},}$$

where:

*t*_1_ and *t*_2_—interference periods of laser 1 and laser 2;

*α*_1_ and *α*_2_—angles of incidence to the normal of the substrate of laser 1 and laser 2: *α*_1_ = 1°; *α*_2_ = 45°.

The refractive index was measured using a two-beam laser interferometer. The laser had a wavelength of (406 ± 0.5) nm. The total error in measuring the refractive index was estimated to be no more than 1.5%.

It should be noted that each refractive index value in the table below corresponds to a separate experiment carried out at a given dew point. After completion of each experiment, the experimental setup was repeatedly prepared for a new cycle of experiments.

## 3. Results and Discussion

**Figure 6:**
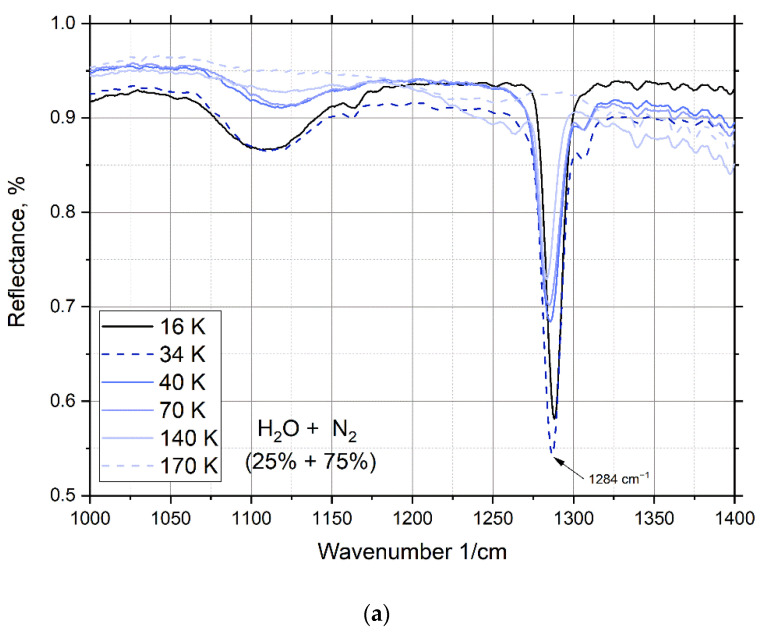

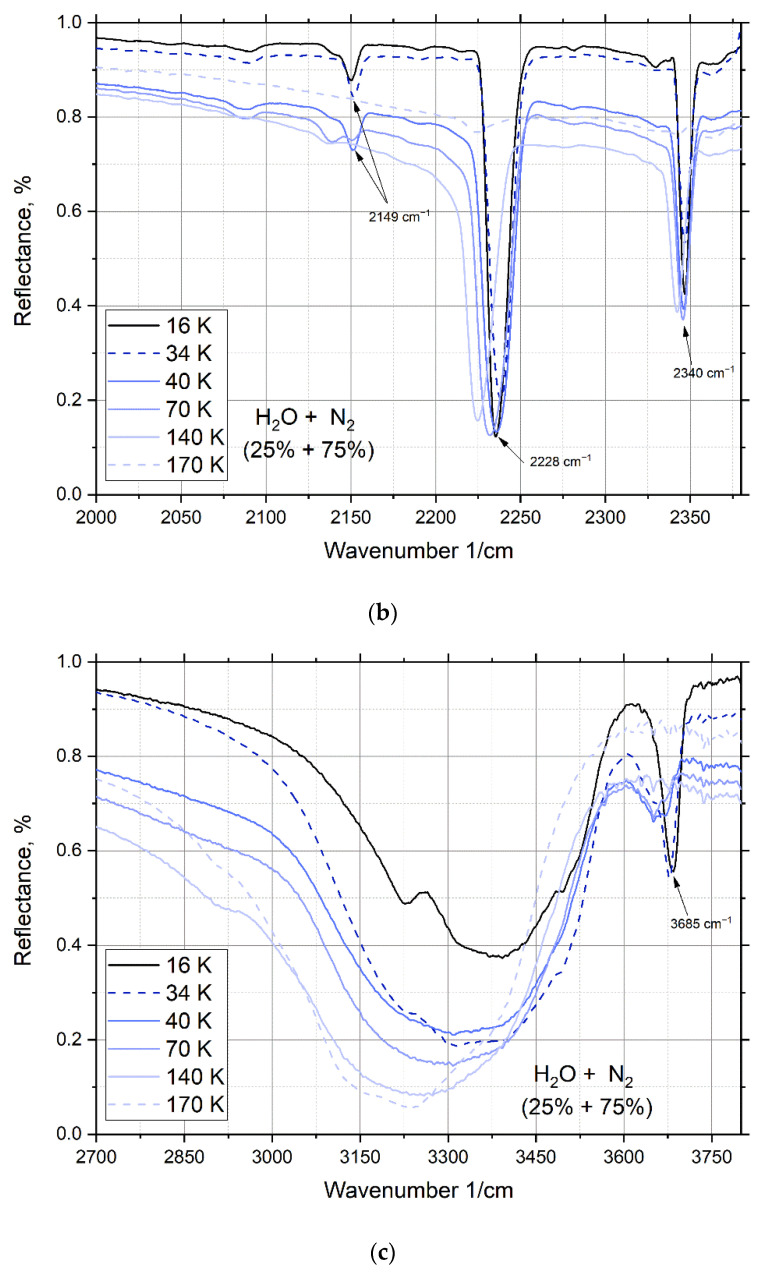
IR spectra of a mixture of water and nitrogen deposited by PVD: (**a**) from 1000 to 1400 cm^−1^; (**b**) from 2000 to 2400 cm^−1^; (**c**) from 2700 to 3750 cm^−1^.

Experiments on the condensation of thin films of mixtures of water-nitrogen and water-argon were carried out at a pressure of 5 μTorr. The mixture was deposited in a cryo-vacuum chamber at a temperature of 16 K, at which a thin film 3.9 µm thick was formed (Figure 5). The film refractive index *n* was 1.293 for water-nitrogen and 1.305 for water-argon. Figure 6 shows the values of the refractive index (RI) versus the deposition temperature of the films of the mixtures under study.

Table 1 presents the results of the obtained refractive indices of RI for pure substances (nitrogen, argon, and water) and mixtures of various concentrations. In general, the admixture of nitrogen increases the RI and adds porosity to the ice structure, which also leads to an increase in the RI. The same is true for ice with an admixture of argon.

The concentration of argon in the mixture has a greater effect on the change in the refractive index than the deposition temperature. 

**Figure 7:**
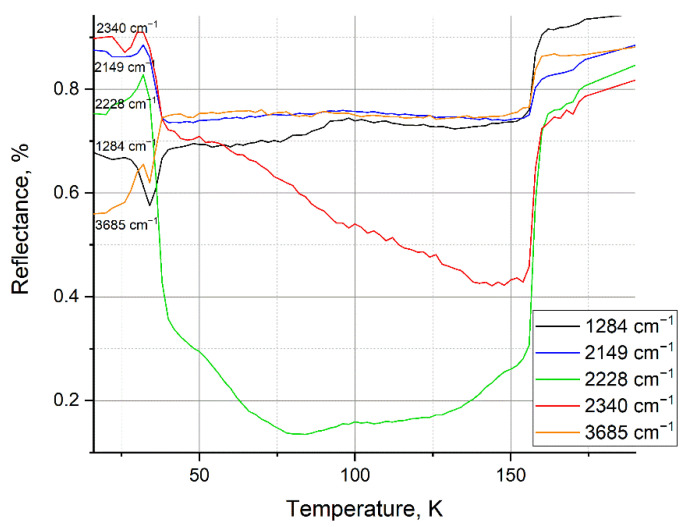
Thermogram for a mixture of water and nitrogen condensed at 16 K.

**Figure 8:**
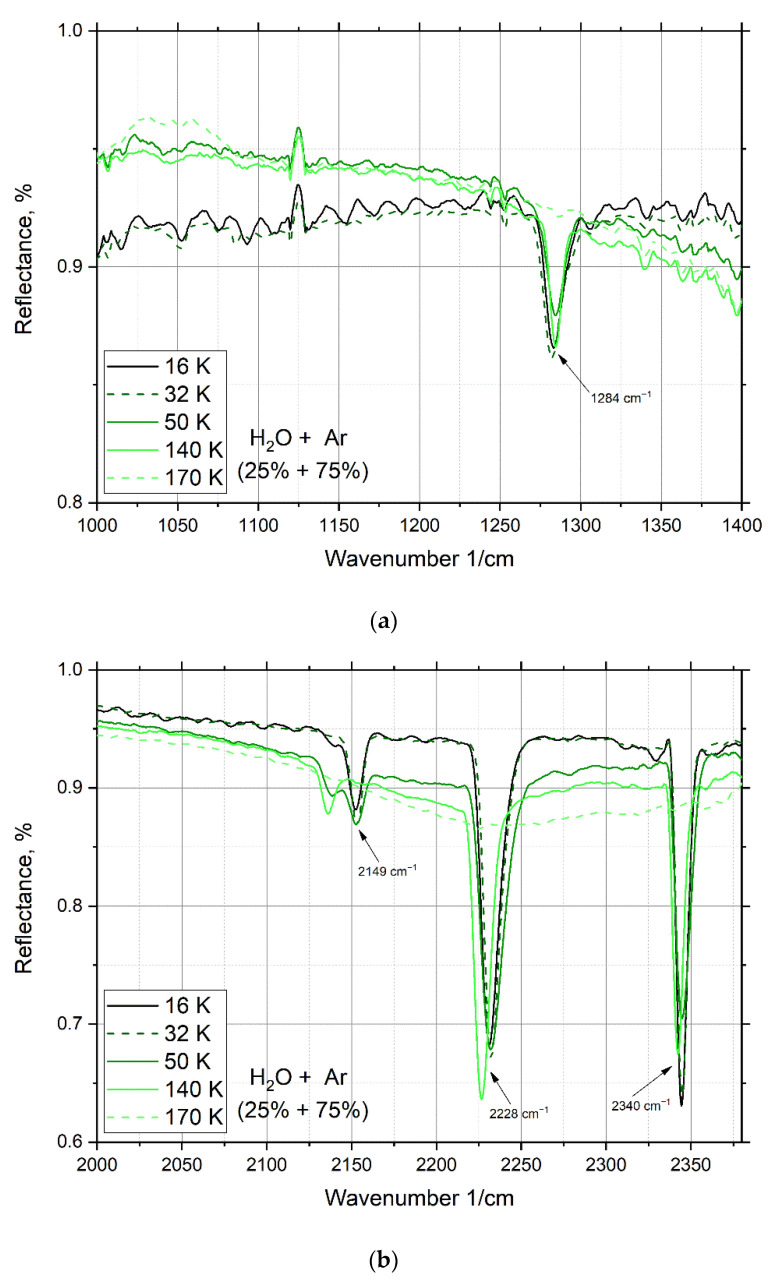

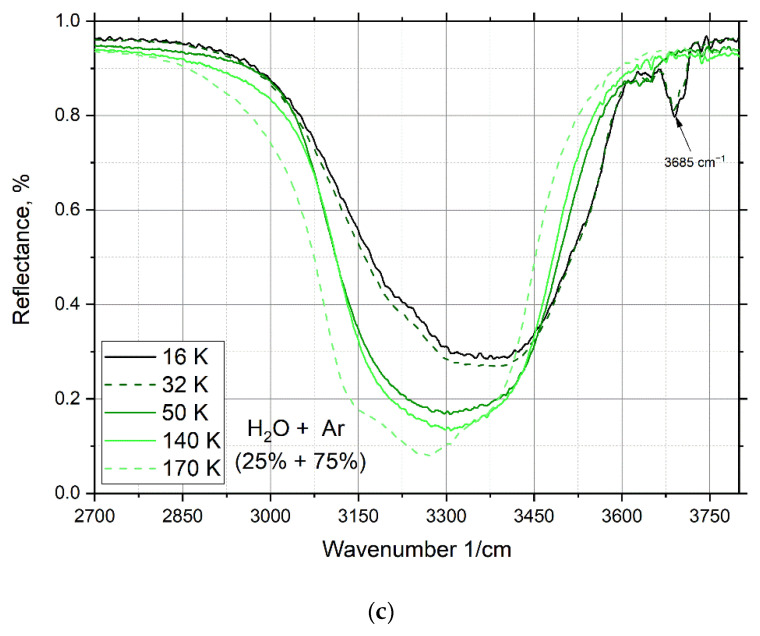
IR spectra of water and argon mixture deposited by PVD method: (**a**) from 1000 to 1400 cm^−1^; (**b**) from 2000 to 2400 cm^−1^; (**c**) from 2700 to 3750 cm^−1^.

**Figure 9:**
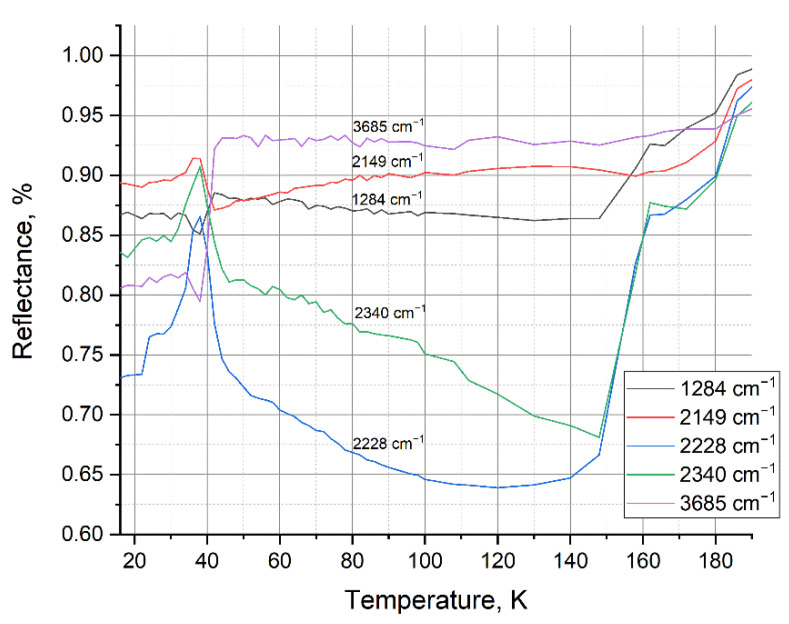
Thermogram for a mixture of water with argon condensed at 16 K.

After the mixture is deposited onto the substrate, the IR spectrum of the film is measured every 2 K during heating (Figure 6, Figure 7, Figure 8 and Figure 9). The figures show the spectra at condensation 16 K, after heating to recondesation 50 K, and upon further heating of the recondensate film. The spectra show a spectral effect, more precisely, dangling spectral bonds and unstable peaks that disappear with a change in the temperature of the clathrate.

To compare the IR spectra and determine the effect of concentration on different modes of the spectrum, the amount of water after the sublimation of nitrogen and argon should be the same for different concentrations of the mixture. For this purpose, a series of experiments were carried out with different concentrations of water content in the mixture (25%, 45%, 75%) in comparison with a pure water sample.

At a water concentration of 25%, peaks appear (Figure 6a—1284 cm^−1^; Figure 6b—2149, 2228, and 2340 cm^−1^), which disappear after heating to 170 K. In Figure 2, the mass spectrum is recorded in the chamber, which confirms which substance creates this peak. As can be seen from Figure 2a, after heating the sample contains only water, and nitrogen is completely sublimated at a temperature of 171.6 K. This proves that these peaks are formed under the action of nitrogen in the composition of the mixture. 

In Figure 6c there is a peak in the region of vibrational vibrations of water molecules (3685 cm^−1^), which begins to disappear when the cryofilm is heated from 42 K. At temperatures above 50 K, the peak is completely smoothed out and disappears, but as mentioned earlier, the nitrogen content of a small concentration is retained up to a temperature of 171.6 K.

Figure 7 shows the thermograms of the previously mentioned peaks for a mixture of 25% water and 75% nitrogen. From 45 K, the process of nitrogen sublimation from the sample begins and continues until its complete release at 171.6 K. The change in the reflection intensity of the IR spectrum occurs in the range from 42 K to 156 K, which indicates a structural transformation inside the sample as nitrogen is released. 

**Figure A1:**
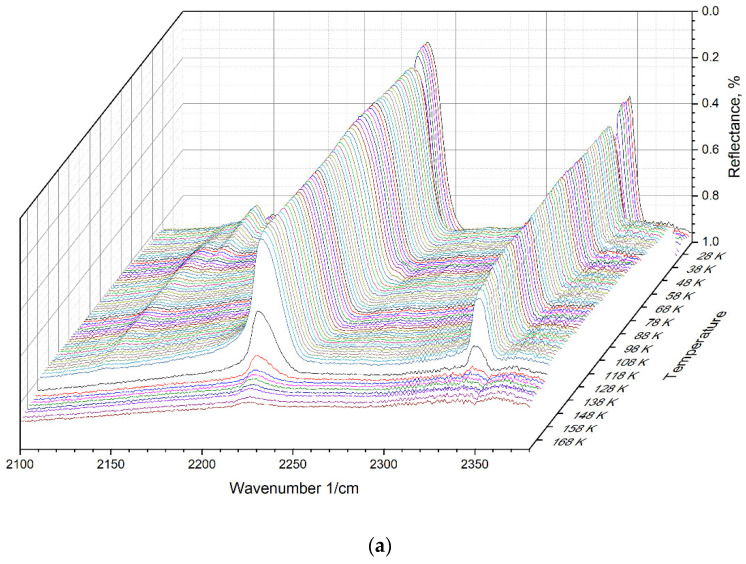

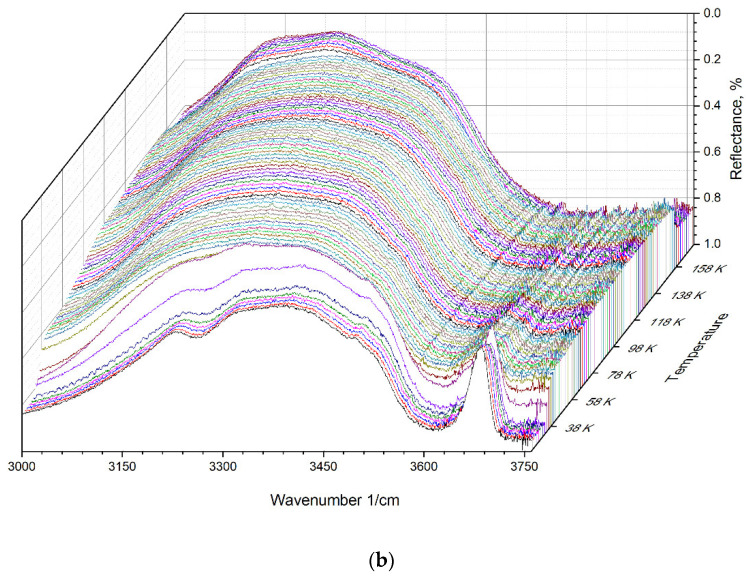
3D IR spectra of a mixture of water and nitrogen deposited by PVD method in the temperature range from 20 to 165 K: (**a**) from 2100 to 2400 cm^−1^; (**b**) from 3000 to 3750 cm^−1^.

Full 3D IR spectra for a mixture of water and nitrogen, obtained every 2 K during the heating process, are presented in Appendix A, Figure A1. 

We have carried out similar experiments for CO_2_ with water. There is also a peak at 3650 cm^−1^, together with the modes of CO_2_ vibrations at 3600 and 3700. However, the peak at 3650 cm^−1^ persists to temperatures above 110 K and disappears only when the guest molecule leaves the film, which does not happen for argon and nitrogen.

Another author’s study [8] mentioned the peak at 2340 cm^−1^ (Figure 8), and it can be added that this peak disappears before the water evaporates and is present in various mixtures of water with argon, nitrogen, carbon dioxide, and in a mixture of {CCl_4_} and argon. 

For a mixture of water and argon, the same spectral effect is observed (a peak that is present during condensation and disappears with heating of the cryovacuum film sample). Peaks at 1284, 2149, 2228, and 2340 cm^−1^ (Figure 8a,b) are also present in the spectrum of the mixture of water and argon, but are less intense than those of the mixture with nitrogen. As with nitrogen, these peaks disappear with the complete sublimation of argon at 172 K. In Figure 8c, for a mixture of water (25%) and argon (75%), a peak appears at wavenumber 3685 cm^−1^, which disappears when the film is heated to 50 K. However, the mass spectrum fixes (Figure 2b) the presence of argon in the cryofilm of the sample up to a temperature of 172 K.

The thermogram of a water and argon mixture in the temperature range from 20 to 180 K (Figure 9) shows changes in the main observed peaks of the IR spectrum. Figure 9 shows the process of sublimation of argon from the sample in temperature range from 45 to 172 K. The temperature range of reflection intensity change and structural transition is from 42 to 160 K. 

**Figure A2:**
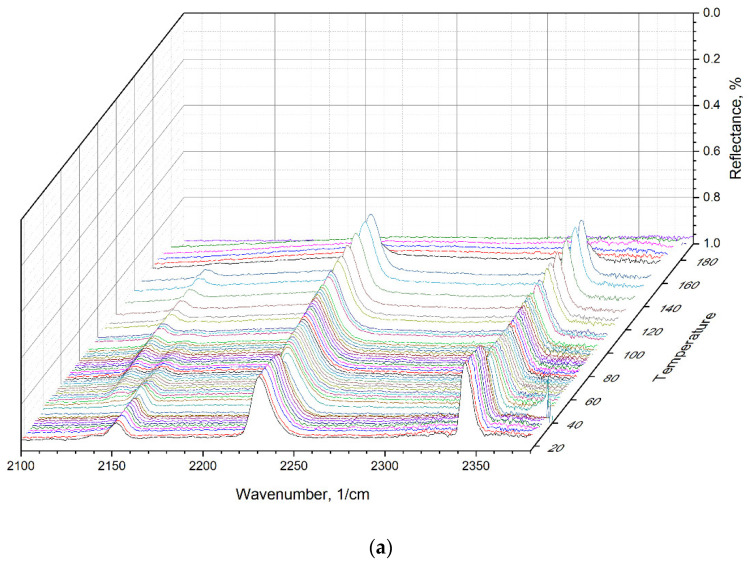

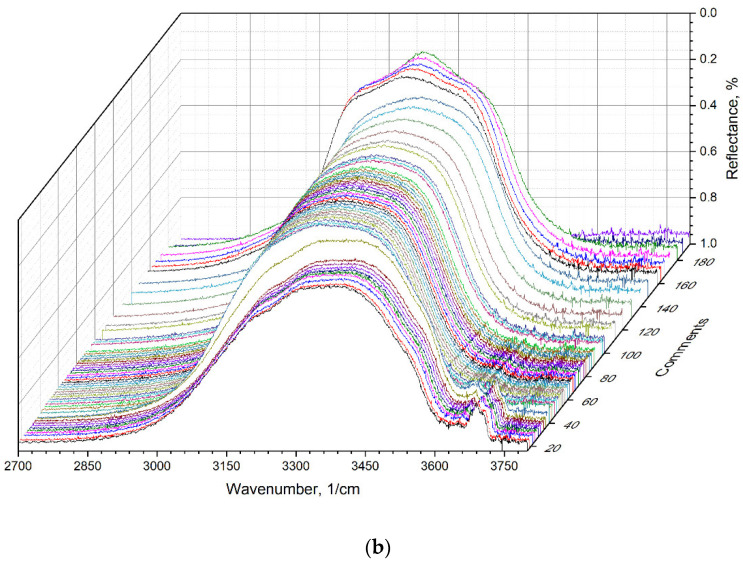
3D IR spectra of mixture water with argon deposited by PVD method in the temperature range from 20 to 180 K: (**a**) from 2100 to 2400 cm^−1^; (**b**) from 2700 to 3750 cm^−1^.

The dependence of the IR spectrum on temperature from 20 to 180 K for a mixture of water and argon is presented in Appendix A, Figure A2.

**Figure 10:**
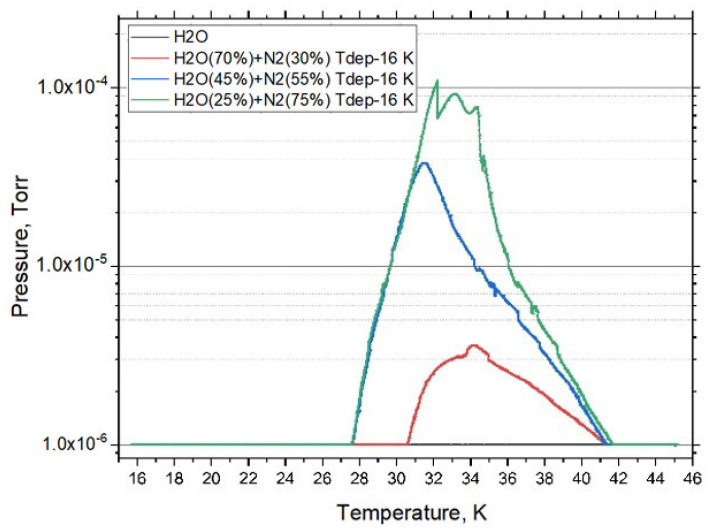
Changes in the pressure in the chamber when nitrogen of various concentrations escapes from the film.

As mentioned earlier, a series of experiments were conducted to determine the influence of mixture concentrations on the escape of nitrogen from the film. Figure 10 shows how the concentration of the mixture affects the change in pressure in the chamber during the first escape of nitrogen from the film during heating. For mixture concentrations (water (45%) + nitrogen (55%) and water (25%) + nitrogen (75%)), the pressure in the chamber increases as the temperature rises to 28 K. As the concentration in the mixture decreases, the onset of nitrogen escape shifts to 31 K (water (70%) + nitrogen (30%)). For all mixture concentrations, most of the nitrogen is sublimed from the sample at 42 K, but not all; the remaining nitrogen leaves the cryofilm sample at 172 K. 

**Figure 11:**
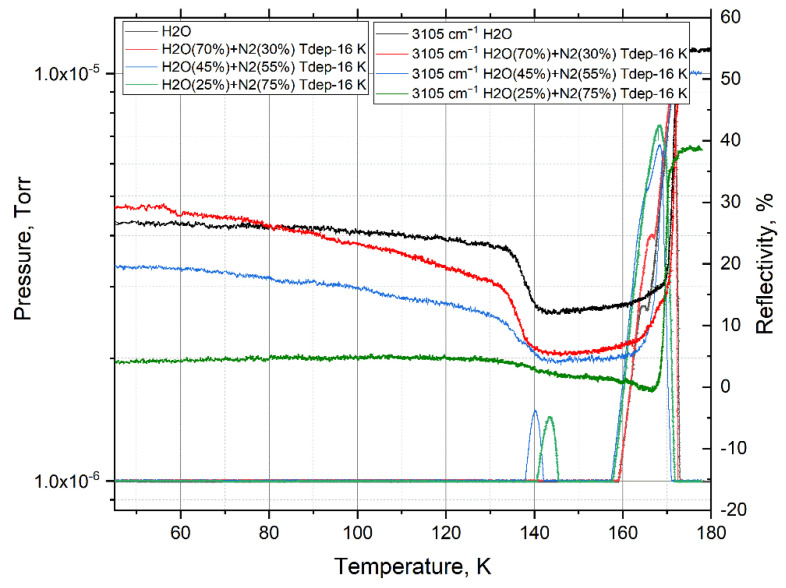
Thermogram of the OH vibration amplitudes and pressure in the chamber with an increase in the temperature of films of various concentrations of a mixture of water with nitrogen.

During the defrosting process at a temperature of 172 K, the remaining nitrogen in the film completely leaves the film (Figure 11). In this case, the process of nitrogen release is more intense the lower the concentration of water (less than 45%) in the mixture. This is confirmed by an increase in the vibration amplitude of the OH bond, which may mean deformation or rupture of the hydrogen bond in water molecules.

## 4. Conclusions

Firstly, the peak that was obtained at a frequency of 3685 cm^−1^ is associated with the presence of water polyaggregates of different compositions formed in the nitrogen matrix during condensation. This process could occur either in the adsorbed layer immediately before condensation or in the nitrogen matrix during the diffusion of water molecules.

When water molecules in clathrates form an internal network of hydrogen bonds, there are several free O-H groups on the surface that do not participate in any hydrogen bonds. Obviously, the key parameters describing these systems are the interactions of internal hydrogen bonds and N_2_-water (Ar-water) interactions. Of particular interest is the change in O-H vibrational modes during N_2_ or Ar binding. An optical study showed that the destruction of the cryofilm structure affects the same vibration frequencies for a water-nitrogen mixture and a water-argon mixture. 

Secondly, a change in the temperature of the sample has a weak effect on the frequency of the combination (bending + libration) type of vibrations (2149 cm^−1^). A strong influence during the destruction of the sample structure is present at the frequencies 1284 cm^−1^ (bending vibration), 2228 and 2340 cm^−1^ (valence multiple bonds with the nitrogen or argon atom). Amorphous ice turns into crystalline ice as the temperature rises, while crystalline ice remains stable as the temperature falls. This can be achieved by considering both the profile of the feature of dangling O-H bonds present in porous amorphous ice and on the surface of large accumulations of crystalline ice, and the profile of the vibrational band of adsorbed particles of nitrogen and argon on the frequency 3685 cm^−1^.

Thirdly, the admixture of nitrogen increases the RI and adds porosity to the ice structure, which also leads to an increase in the RI. The concentration of argon in the mixture has a greater effect on the change in the refractive index than the deposition temperature.

Dangling bonds in IR spectra near 3700 cm^−1^ are a source of information about the structural properties of amorphous water ice and especially icy mixtures of water and other frozen gases. A similar spectral effect was found by our authors in a series of other experiments conducted at the Laboratory of Cryophysics and Cryotechnology at Al-Farabi Kazakh National University for various concentrations of mixtures of water with nitrogen, methane, ethanol, CO_2_, or CCl_4_ [25,26,27,28,29].

## Figures and Tables

**Table 1:** Dependence of the refractive index (RI) on mixture concentrations and deposition temperature.

Substance	Temperature, K	*n*
H_2_O	16	1.19 ± 0.02
H_2_O	20	1.20 ± 0.02
H_2_O	30	1.22 ± 0.02
N_2_	16	1.22 ± 0.02
Ar	16	1.26 ± 0.02
Ar	20	1.27 ± 0.02
Ar	30	1.29 ± 0.02
H_2_O (25%) + N_2_ (75%)	16	1.29 ± 0.02
H_2_O (45%) + N_2_ (55%)	16	1.28 ± 0.02
H_2_O (70%) + N_2_ (30%)	16	1.25 ± 0.02
H_2_O (25%) + Ar (75%)	16	1.32 ± 0.02
H_2_O (48%) + Ar (52%)	16	1.30 ± 0.02
H_2_O (74%) + Ar (26%)	16	1.23 ± 0.02
H_2_O (24%) + Ar (76%)	20	1.30 ± 0.02
H_2_O (50%) + Ar (50%)	20	1.29 ± 0.02
H_2_O (70%) + Ar (30%)	20	1.24 ± 0.02
H_2_O (25%) + Ar (75%)	30	1.31 ± 0.02
H_2_O (48%) + Ar (52%)	30	1.33 ± 0.02
H_2_O (74%) + Ar ( 26%)	30	1.24 ± 0.02

## Data Availability

The data presented in this study are available on request from the corresponding author.
